# Coronavirus Disease 2019-Related Alterations of Total and Anti-Spike IgG Glycosylation in Relation to Age and Anti-Spike IgG Titer

**DOI:** 10.3389/fmicb.2022.775186

**Published:** 2022-04-15

**Authors:** Christian Schwedler, Marta Grzeski, Kai Kappert, Jörn Rust, Guido Heymann, Berthold Hoppe, Véronique Blanchard

**Affiliations:** ^1^Institute of Diagnostic Laboratory Medicine, Clinical Chemistry and Pathobiochemistry, Charité – Universitätsmedizin Berlin, Corporate Member of Freie Universität Berlin, Humboldt-Universität zu Berlin, Berlin, Germany; ^2^German Cancer Consortium (DKTK), German Cancer Research Center (DKFZ), Heidelberg, Germany; ^3^Labor Berlin – Charité Vivantes GmbH, Berlin, Germany; ^4^Department of Anaesthesiology, Critical Care, and Pain Medicine, BG Klinikum Unfallkrankenhaus Berlin, Berlin, Germany; ^5^Institute of Laboratory Medicine, BG Klinikum Unfallkrankenhaus Berlin, Berlin, Germany

**Keywords:** IgG glycosylation, *N*-glycopeptides, glycans, Spike, SARS-CoV-2, COVID-19, MALDI-TOF

## Abstract

The coronavirus disease 2019 (COVID-19) caused by the severe acute respiratory syndrome coronavirus-2 (SARS-CoV-2) has been affecting the world since January 2020 and has caused millions of deaths. To gain a better insight into molecular changes underlying the COVID-19 disease, we investigated here the *N*-glycosylation of three immunoglobulin G (IgG) fractions isolated from plasma of 35 severe COVID-19 patients, namely total IgG_1_, total IgG_2_, and anti-Spike IgG, by means of MALDI-TOF-MS. All analyses were performed at the glycopeptide level to assure subclass- and site-specific information. For each COVID-19 patient, the analyses included three blood withdrawals at different time-points of hospitalization, which allowed profiling longitudinal alterations in IgG glycosylation. The COVID-19 patients presented altered IgG *N*-glycosylation profiles in all investigated IgG fractions. The most pronounced COVID-19-related changes were observed in the glycosylation profiles of antigen-specific anti-Spike IgG_1_. Anti-Spike IgG_1_ fucosylation and galactosylation showed the strongest variation during the disease course, with the difference in anti-Spike IgG_1_ fucosylation being significantly correlated with patients’ age. Decreases in anti-Spike IgG_1_ galactosylation and sialylation in the course of the disease were found to be significantly correlated with the difference in anti-Spike IgG plasma concentration. The present findings suggest that patients’ age and anti-S IgG abundance might influence IgG *N*-glycosylation alterations occurring in COVID-19.

## Introduction

Coronaviruses have been responsible for three pandemics this century with high mortality although they are usually responsible for only benign colds. The severe acute respiratory syndrome coronavirus (SARS-CoV) and the Middle East respiratory syndrome coronavirus (MERS-CoV) caused nosocomial outbreaks in 2002 and in 2012, respectively, due to a virus transfer from animals to humans (De Wit et al., 2016; Reis et al., 2021). However, in 2019, the severe acute respiratory syndrome coronavirus-2 (SARS-CoV-2) emerged worldwide as the causative agent of the coronavirus disease 2019 (COVID-19), which can range from a mild disease course to pneumonia requiring hospitalization and to life-threatening multi-organ failure in the most severe cases. So far, around 431 million cases and almost 6 million deaths have been reported worldwide.

Immunoglobulins G (IgG) are the most abundant antibodies present in human blood with concentrations ranging from 7 to 18 mg/ml. Produced by mature plasma cells, they play a crucial role in the regulation of inflammation and immune reactions in humans and animals (Karsten et al., 2012; Hess et al., 2013; Bartsch et al., 2020). In humans, IgG is subdivided into four subclasses named according to their abundance: IgG_1_, IgG_2_, IgG_3_, and IgG_4_ (Schur, 1988). Although they share about 90% of amino acid homology, each subclass differs with respect to effector functions and binding affinities toward Fc gamma receptors (FcγRs) (Wang and Ravetch, 2019). Each IgG molecule consists of two parts: fragment antigen binding (Fab) that is sporadically glycosylated and fragment crystallizable (Fc), which is glycosylated at asparagine (Asn) 297 within each heavy chain. IgG glycosylation at Asn297 modulates its interactions with Fcγ receptors (FcγRs). In particular, absence of core-fucosylation was shown to tremendously increase the binding to FcγRIIIa and enhance antibody-dependent cellular cytotoxicity. This principle is used in the production of recombinant monoclonal antibodies by the pharmaceutical industry for anti-cancer therapy (Yu et al., 2017).

IgG glycosylation at Asn297 consists of complex-type biantennary *N*-glycans being highly fucosylated, slightly sialylated and having various degrees of galactosylation (Clerc et al., 2016). IgG glycosylation is age- as well as sex-dependent (Bakovic et al., 2013; Stambuk et al., 2020) and has been widely studied in health and disease. Fucosylation is relatively stable in adulthood and upon aging (Dall’olio et al., 2013; De Haan et al., 2016). On the opposite, sialylation and galactosylation decrease with age (Dall’olio et al., 2013) because their occurrence is related to estrogen regulation both in men and women (Ercan et al., 2017). Moreover, the latter glycosylation features are also gender-dependent: IgG galactosylation and sialylation are higher in pre-menopausal women as compared with men (Bakovic et al., 2013). After menopause, sialylation, and galactosylation levels are similar in men and women (Bakovic et al., 2013). Distinct IgG glycosylation features are also associated with a broad range of inflammatory pathologies. Precisely, IgG glycosylation is modulated during chronic as well as acute inflammation. In rheumatoid arthritis patients, decreased galactosylation was correlated with clinical parameters (Van De Geijn et al., 2009; Schwedler et al., 2018) and with decreased activity of galactosyltransferase in plasma B-cells (Axford et al., 1987). During bacterial and viral infections, decreased IgG galactosylation was observed for hepatitis B and tuberculosis (Pilkington et al., 1995; Ho et al., 2015; Irvine and Alter, 2020).

In COVID-19 patients, however, regulation of glycosylation has not been addressed in detail. Thus, in order to gain a better understanding of IgG glycosylation upon SARS-CoV-2 infection, we studied total IgG and anti-SARS-CoV-2 IgG glycosylation at Asn297 in a cohort of patients hospitalized in Berlin (Germany) during disease course.

## Materials and Methods

### Sample Collection

The study was approved by the Institutional Review Board at Charité – Universitätsmedizin Berlin, Campus Virchow-Klinikum, Germany (no. EA2/095/20) and at the Ärztekammer Berlin, Germany (Eth-23/20). All experiments were performed in accordance with relevant guidelines and regulations. Additional written informed consent was taken for Eth-23/20. The investigated cohort consisted of 35 COVID-19 patients hospitalized Charité – Universitätsmedizin Berlin or Unfallkrankenhaus Berlin between 31.03.2020 and 31.12.2020 and 35 age- and sex-matched healthy controls (HC) (Table 1). For each COVID-19 patient, the analysis included three plasma samples, each withdrawn at a different time-point of the hospital stay. Blood withdrawal was performed according to the standard of care and plasma was separated by centrifugation at 2200 × *g* for 10 min using plasma separation tubes with polymer gel and lithium heparin (Becton Dickinson, Medical-Pharmaceutical System, Franklin Lakes, NJ, United States, or Greiner Bio-One, Kremsmünster, Austria). Obtained plasma was aliquoted and stored at −80°C until the time of further analysis. C-reactive protein (CRP) was determined by immunoturbidimetry.

**TABLE 1:** Demographics of the cohorts used in this study.

		COVID-19 patients		
Parameter	HC	First detection	Middle detection	Last detection
*n*	35	35	35	35
Age, years	65.0 (53.2–75.6)	64.0 (55.6–75.8)		
Female sex, *n* (%)	14 (40.0)	14 (40.0)		
Hospital stay, days		2 (0–6)	12 (5–17)	20 (13–30)
ICU, *n* (%)		28 (80.0)	24 (68.6)	7 (20.0)
Anti-S IgG, positive (%)		27 (77.1)	29 (82.9)	34 (97.1)
Anti-S IgG, IU		25.4 ± 33.0 (0.1–128.4)	77.3 ± 143.9 (0.1–721.1)	61.7 ± 68.5 (0.1–322.4)
CRP, mg/L		83.5 ± 86.9 (0.6–320.5)	47.8 ± 54.3 (0.6–284.7)	44.7 ± 59.4 (0.6–235.3)

*HC, healthy controls; ICU, intensive care unit; CRP, C-reactive protein; Anti-S IgG, anti-SARS-CoV-2 immunoglobulin G; IU, international unit. Age and hospital stay are shown as median (interquartile range), whereas CRP and anti-S IgG are shown as mean ± SD (range).*

### Enzyme-Linked Immunosorbent Assay

Plasma anti-SARS-CoV-2-IgG levels were semi-quantified using a commercially available enzyme-linked immunosorbent assay (ELISA) system [Anti-SARS-CoV-2-ELISA (IgG), EUROIMMUN Medizinische Labordiagnostika AG, Lübeck, Germany]. The system consists of immobilized recombinant SARS-CoV-2 Spike S1 subunit protein, which is specifically recognized and bound by its cognate anti-SARS-CoV-2 Spike S1 IgGs (in the following parts of this work referred to as anti-Spike/anti-S IgGs) contained in COVID-19 positive samples. The binding efficiency was determined based on the value of an extinction sample to extinction calibrator ratio, a relative measure of the anti-S IgG concentration in plasma. Plasma samples with anti-S IgG IU > 1.1 were assessed as COVID-19 positive and those with IU > 4.0 were used for anti-S IgG glycosylation analysis.

### Purification of Anti-S IgG From Plasma

A 20-μl aliquot of each COVID-19 patient plasma sample was diluted 1:50 in the sample buffer supplied with the ELISA kit. The resulting mixture was distributed among four consecutive wells of the anti-SARS-CoV-2-ELISA plate and incubated for 1 h at 37°C. Afterward, the supernatants were discarded and the wells were washed with 3 × 300 μl of wash buffer (supplied with the ELISA kit) and 2 × 300 μl of Milli-Q water. The retained anti-S IgG antibodies were eluted with 3 × 100 μl of 100 mM formic acid. For each plasma sample, the eluates from all four wells were pooled together and evaporated in a vacuum centrifuge.

### Purification of Total Immunoglobulins G From Plasma

Total IgG antibodies were isolated from human plasma as described previously (Schwedler and Blanchard, 2019). Briefly, a 5-μl aliquot of each plasma sample was incubated in a 96-well filter plate (AcroPrep Advance filter plate, 1.2 μm Supor membrane, Pall Life Sciences, Dreieich, Germany) with Protein A Sepharose beads (GE Healthcare, Uppsala, Sweden) for 1 h at 37°C. The beads were washed thoroughly with 1 × PBS and Milli-Q water under vacuum (multi-well plate vacuum manifold, Pall Life Sciences). Afterward, the captured IgG molecules (IgG_1_, IgG_2_, and IgG_4_) were eluted with 2 × 50 μl of 100 mM formic acid, dried by vacuum centrifugation, and stored at −20°C until further use.

### Tryptic Digestion and Glycopeptide Purification

Tryptic digestion of IgG followed by glycopeptide purification was performed as described previously (Wieczorek et al., 2020). Briefly, dried antigen-specific anti-S IgG or total IgG fractions were dissolved in 50 μl of 50 mM ammonium bicarbonate (Merck, Darmstadt, Germany). Sequencing grade modified trypsin (Promega, Madison, WI, United States) was reconstituted to a concentration of 0.2 μg/μl in a buffer provided by the manufacturer and 5 μl was added to each sample. After overnight incubation at 37°C, the digested IgGs were dried by vacuum centrifugation and stored at −20°C until further processing.

Immunoglobulins G glycopeptide enrichment was achieved using self-made cotton-HILIC microcolumns (Selman et al., 2011), conditioned with 3 × 50 μl of Milli-Q water and 3 × 50 μl of 85% ACN. Then, the trypsinized IgG samples were resuspended in 50 μl of 85% ACN and applied to the microcolumns. The columns were washed with 3 × 50 μl of 85% ACN containing 0.1% TFA and 3 × 50 μl of 85% ACN. Eventually, retained IgG glycopeptides were eluted with 6 × 50 μl of Milli-Q water, dried in a vacuum centrifuge, and stored at −20°C until MALDI-TOF-MS measurements.

### MALDI-TOF Measurements and Data Analysis

Dried total and anti-S IgG glycopeptides were dissolved in 70 and 5 μl Milli-Q water, respectively. Of these, 1 μl was spotted on the stainless steel MALDI target plate (Bruker Daltonics, Bremen, Germany). After drying, each spot was overlaid with 1 μl of 2.5 mg/ml 4-chloro-α-cyanocinnamic acid (ClCCA, Sigma Aldrich, St. Louis, MO, United States) in 70% ACN and was left to air-dry at room temperature. Measurements were performed on Ultraflex III mass spectrometer (Bruker Daltonics, Bremen, Germany) equipped with Smart Beam Laser (laser frequency 100 Hz). Calibration was performed with Peptide Calibration Standard II (Bruker Daltonics, Bremen, Germany). Mass spectra were recorded in reflectron negative ionization mode using the *m/z* range of 1000–5000 and a partial “random-walk” laser movement mode. All IgG glycopeptides were detected as [M-H]^–^ species and are listed in Supplementary Table 1. The recorded mass spectra were exported as ASCII text files, and the subsequent data processing including re-calibration, baseline subtraction, and peak extraction was performed using the MassyTools software (Jansen et al., 2015). The re-calibration of total IgG_1_/anti-S IgG_1_ and total IgG_2_ mass spectra was performed using the list of six IgG_1_ and six IgG_2_ glycopeptides (G0F, G1F, G0FN, G2F, G1FN, and G2FS1), respectively. The intensities of the detected glycopeptides were normalized for total IgG_1_, total IgG_2_, and anti-S IgG_1_. Afterward, the subclass-/type-specific IgG glycosylation profiles were represented in a form of four glycosylation traits, i.e., fucosylation, galactosylation, sialylation, and bisection, determined by summing up relative intensities of respective glycopeptide structures as described below:

Fucosylation (Fuc) = G0F + G1F + G2F + G0FN+ G1FN + G2FN + G1FS1 + G2FS1 + mono G0F+ mono G1F;

Galactosylation (Gal) = (G1F + G1FN + G1FS1+ Mono G1F + G1 + G1N) * 0,5 + G2F + G2FN + G2FS1 + G2 + G2N + G2S1;

Sialylation (Sial) = G1FS1 + G2FS1 + G1S1 + G2S1;

Bisecting GlcNAc (Bisec) = G0FN + G1FN + G2FN + G0N + G1N + G2N.

It should be noted that, in the case of IgG_2_, fucosylation could not be determined due to the mass overlap of its afucosylated structures with the major glycopeptides of the IgG_4_ subclass.

### Statistical Analysis

Statistical analyses were performed using IBM SPSS version 25.0 (IBM, Armonk, NY, United States) and PRISM 6.0 software (GraphPad Software, La Jolla, CA, United States). Two-way ANOVA was performed to test whether total IgG_1_-, anti-S IgG_1_-, and total IgG_2_-specific glycosylation traits change over the course of the COVID-19 disease. Wilcoxon signed-rank test was used to determine whether total IgG_1_ and anti-S IgG_1_ glycosylation profiles in COVID-19 patients differ between the first and the last time-point of hospitalization. Mann–Whitney *U*-test was used to determine whether total IgG_1_ glycosylation differs between COVID-19 patients and HC. Association between the length of COVID-19 patients’ hospital stay and age was evaluated by Pearson’s correlation. Subsequently, the same statistical tests were performed to determine whether COVID-19-related differences in total IgG_1_, total IgG_2_, and anti-S IgG_1_ glycosylation correlate with patients’ age and with the difference in plasma CRP and anti-S IgG concentration recorded in the course of the disease. For each parameter/glycosylation trait, the difference between the last and the first time-point of hospitalization (Δ) was calculated according to the formula: ΔX = X last − X first. To control the Type I Error, all individual *p*-values were adjusted employing the Bonferroni correction method (*p*-values were multiplied by the corresponding number of tests). Eventually, the Bonferroni corrected *p*-values are indicated as *p < 0.05, **p < 0.01, ***p < 0.001.

## Results

In this study, we investigated the glycosylation profiles of total IgG_1_, total IgG_2_, and antigen-specific anti-S IgG_1_ isolated from plasma samples of COVID-19 patients by means of MALDI-TOF-MS. The investigated cohorts consisted of 35 severe COVID-19 patients and 35 sex- and age-matched HC, whose demographics are presented in Table 1. A majority of COVID-19 patients (80%) was treated at intensive care unit (ICU) wards. The hospitalization duration varied from 4 to 57 days (median: 20 days) and a total of 33 patients survived the COVID-19 infection. For each COVID-19 patient, three plasma samples (referred to as first, middle, and last) were analyzed, each of which corresponded to a different time-point of patient’s hospital stay, namely the beginning, middle, and the end. The first anti-S IgG glycopeptide detection corresponded to hospitalization day 0–6 and, with few exceptions, matched with the first day when anti-S IgG could be unambiguously detected (≥1.1 IU). Altogether, the investigated material consisted of 140 plasma samples (HC: 35; COVID-19 patients: 105) resulting in 245 IgG glycopeptide samples (total IgG: 140; anti-S IgG: 105) being measured.

**FIGURE 1:**
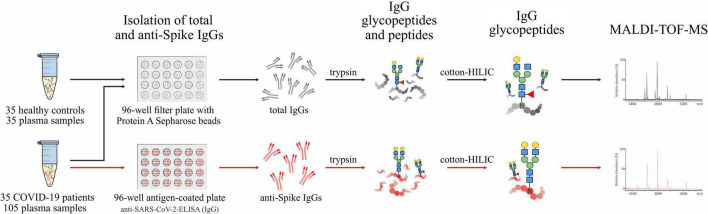
Schematic representation of the analytical workflow used in this study.

The analytical workflow used in this study is presented in Figure 1. Inter-day repeatability of the sample preparation was verified by analyzing the same plasma sample in triplicate on three consecutive days. The results of repeatability testing are presented in Supplementary Figure 1. The mean coefficient of variation values were 3.11 for total IgG_1_, 3.75 for total IgG_2_, and 4.71 for anti-S IgG_1_, indicating a very good repeatability of the applied method.

**FIGURE 2:**
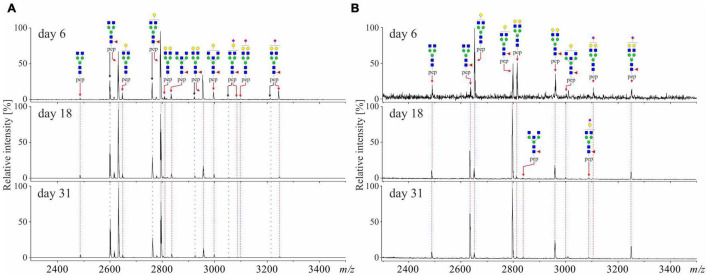
Representative MALDI-TOF-mass spectra [M-H]^–^ of **(A)** total IgG_1/2_ and **(B)** anti-Spike IgG_1_ isolated from plasma of severe COVID-19 patient (male, age 51) at beginning (day 6), midpoint (day 18), and the end (day 31) of the disease. IgG_1_ glycopeptides are marked in red and IgG_2_ glycopeptides are marked in black.

The representative MALDI-TOF mass spectra [M-H]^–^ of total and anti-S IgG glycopeptide fractions are presented in Figure 2. In the total IgG fraction, up to 28 glycopeptide signals were detected, of which 17 corresponded to IgG_1_ and 11 to IgG_2_ subclass (Figure 2A and Supplementary Table 1). Five IgG_2_ structures (i.e., G1, G2, G1N, G2N, and G2S1) could not be unambiguously assigned due to the mass overlap with IgG_4_ glycopeptides and hence were not included in the analysis. It should be noted that the anti-S IgG fraction contained solely IgG_1_ glycopeptides (Figure 2B); this is why it is referred to as anti-S IgG_1_ in the following parts of this work. As shown in Figure 2B for one representative COVID-19 patient, at the beginning of the disease (hospitalization day 6), the most abundant anti-S IgG_1_ glycopeptides are the afucosylated G1 and G2 structures, whereas the fucosylated G0F and G1F glycopeptides become the most abundant structures later in the disease course (day 18 and day 31).

Aiming at performing statistical comparisons between clinical parameters and glycosylation features, the glycosylation profiles of total and anti-S IgGs were represented in the form of four glycosylation traits, namely fucosylation, galactosylation, sialylation, and bisection, calculated separately for each IgG subclass/type, as described in section “MALDI-TOF Measurements and Data Analysis.” The relative abundance and SD values of all total IgG_1_, total IgG_2_, and anti-S IgG_1_ glycosylation traits and individual glycopeptide structures detected in HC and COVID-19 patients are presented in Supplementary Table 2.

**FIGURE 3:**
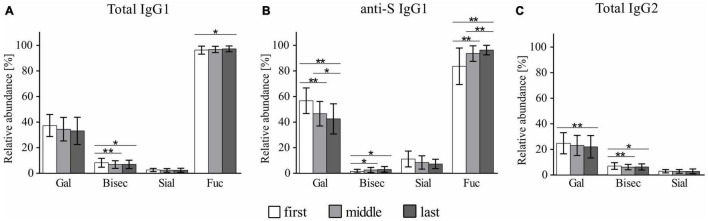
Profiles of COVID-19-related glycosylation alterations in **(A)** total IgG_1_, **(B)** anti-S IgG_1_, and **(C)** total IgG_2_. *p < 0.05, **p < 0.01. The calculation of the glycosylation traits Gal, Bisec, Sial, and Fuc is given in section “MALDI-TOF Measurements and Data Analysis.”

As visible in Figure 3, COVID-19 was found to be associated with significant changes in total IgG_1_, total IgG_2_, and antigen-specific anti-S IgG_1_ glycosylation. In general, all investigated IgG fractions presented similar patterns of COVID-19-related glycosylation alterations, marked by a gradual decrease in galactosylation and sialylation and a concomitant gradual increase in fucosylation (in the case of IgG_2_, fucosylation was not determined). Bisection was the only glycosylation trait that showed a distinct profile of COVID-19-related alterations in total IgG_1_/total IgG_2_ and antigen-specific anti-S IgG_1_ antibodies, in which it, respectively, decreased and increased in the course of the disease. IgG_2_ was found to be marked by an overall lower galactosylation compared to both IgG_1_ fractions, which seems to be a universal feature of IgG_2_ glycosylation and is in line with previous reports (Bakovic et al., 2013; Wieczorek et al., 2020). Notably, the strongest COVID-19-related glycosylation alterations were recorded for antigen-specific anti-S IgG_1_. Among all glycosylation traits, anti-S IgG_1_ fucosylation and galactosylation had a particularly high variation in the course of the disease, with anti-S IgG_1_ fucosylation (first: 83.3%; last: 95.9%) showing a 12% increase and anti-S IgG_1_ galactosylation (first: 56.4%, last: 42.2%) a 14% decrease. Notably, as visible in Supplementary Figure 2, further stratification of COVID-19 cohort based on the length of hospitalization revealed that the above-described alterations prevail in patients with prolonged hospital stay (≥20 days).

**FIGURE 4:**
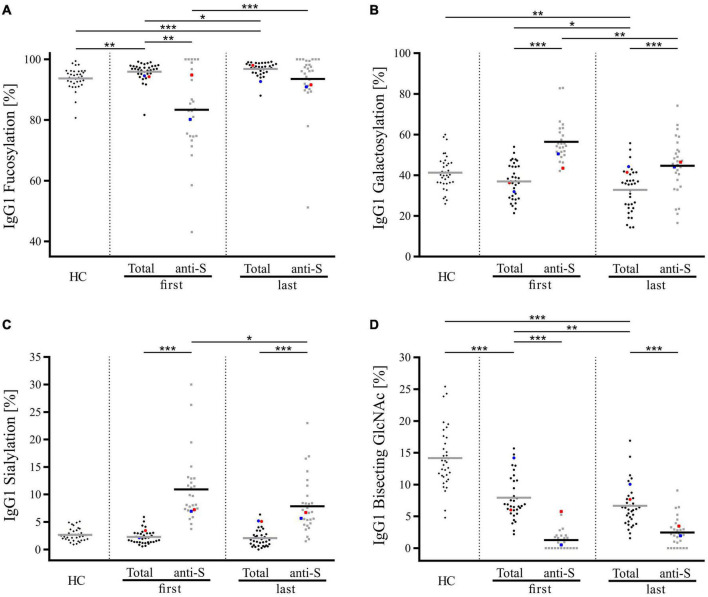
Comparison of Fc glycosylation of total and anti-S IgG_1_ in COVID-19 patients at the beginning and the end of hospitalization and total IgG_1_ Fc glycosylation of healthy controls (HC). **(A)** Fucosylation, **(B)** galactosylation, **(C)** sialylation, and **(D)** bisecting GlcNAc. The calculation of the glycosylation traits Gal, Bisec, Sial, and Fuc is given in section “MALDI-TOF Measurements and Data Analysis.” Data points corresponding to the two deceased COVID-19 patients are indicated in red (patient 1) and blue (patient 2). The Bonferroni corrected *p*-values are indicated as *p < 0.05, **p < 0.01, ***p < 0.001.

Despite similar trends of COVID-19-related alterations, glycosylation profiles of total and antigen-specific anti-S IgG_1_ antibodies were found to differ significantly, with the strongest discrepancy being observed at the beginning of hospitalization. Precisely, as visible in Figure 4, anti-S IgG_1_ antibodies secreted early in the COVID-19 course were marked by significantly lower fucosylation and bisection and significantly higher galactosylation and sialylation as compared to total IgG_1_ of COVID-19 patients. Notably, although anti-S IgG_1_ glycosylation was observed to alter continuously in the disease course (Supplementary Figure 3), statistically significant differences between total and anti-S IgG_1_ were as well detected at the end of hospitalization for the following glycosylation traits: galactosylation, sialylation, and bisection. It is also notable that the profile of total IgG_1_ glycosylation was found to differ significantly between COVID-19 patients and HC (Figure 4). In particular, total IgG_1_ glycopeptides of COVID-19 patients were marked by decreased abundance of bisecting GlcNAc and increased fucosylation both at the beginning and at the end of hospitalization. Contrarily, sialylation of total IgG_1_ was unaltered in COVID-19 patients as compared to HC, whereas galactosylation was significantly decreased in COVID-19 patients at the end of hospitalization.

**FIGURE 5:**
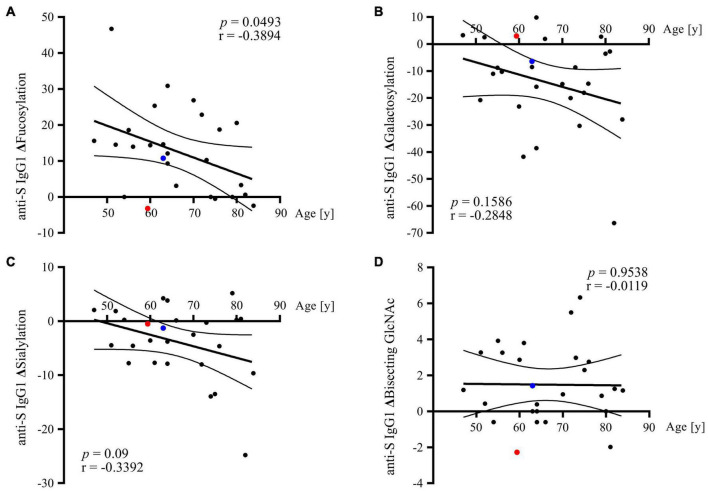
Correlation of anti-S IgG_1_ glycosylation alterations with patients’ age. In each graph, the *Y* axis represents the difference (Δ) in the relative abundance of the respective glycosylation trait recorded between the last and first hospitalization time-point: **(A)** Δfucosylation, **(B)** Δgalactosylation, **(C)** Δsialylation, and **(D)** Δbisecting GlcNAc. Data points corresponding to the two deceased COVID-19 patients are indicated in red (patient 1) and blue (patient 2).

In the investigated COVID-19 cohort, the length of hospitalization was expectedly found to correlate with patients’ age (*p* = 0.038, Pearson *r* = 0.3552). Since, in line with literature data (Gudelj et al., 2018), IgG *N*-glycan composition in both healthy and COVID-19 patients was likewise observed to differ based on patients’ age (Supplementary Figure 4), we next tested whether this age-dependency is also reflected at the level of COVID-19-related glycosylation changes recorded in total IgG_1_, total IgG_2_, and anti-S IgG_1_. Among all investigated antibody fractions, age-dependency of COVID-19-related glycosylation alterations could be detected only in anti-S IgG_1_. The results of the correlation analyses are presented in Figure 5, in which the *X* axes represent patient age, whereas the *Y* axes represent the change (Δ) in the respective anti-S IgG_1_ glycosylation trait recorded between the last and first hospitalization time-points. Notably, anti-S IgG_1_ fucosylation was the only glycosylation trait, in which the difference between the final and the initial level was significantly correlated with patients’ age. Precisely, in the course of COVID-19 disease, younger patients exhibited significantly stronger alteration in anti-S IgG_1_ fucosylation level as compared to older ones. Contrarily, in the case of galactosylation and sialylation, a more prominent change between the final and the initial level was observed in older COVID-19 patients, however, the respective correlations were statistically insignificant.

Since altered IgG glycosylation is a common feature of inflammatory conditions, we next tested whether COVID-19-related total and anti-S IgG_1_ glycosylation alterations correlate with changes in CRP and plasma anti-S IgG concentration in the course of COVID-19. As compared to physiological CRP concentrations that range between 0.8 and 3.0 mg/L (Shine et al., 1981), CRP levels in COVID-19 patients were strongly increased, with the highest levels being expectedly recorded at the beginning of hospitalization (except for the two patients who did not survive COVID-19) (Supplementary Figure 5A). In the course of patients’ hospitalization, the CRP and anti-S IgG levels were observed to, respectively, decrease and increase in COVID-19 patients; however, for none of these parameters the difference between the final and the initial level was correlated with patients’ age (Supplementary Figures 5C,F). As visible in Table 2, Pearson’s correlation analyses revealed that COVID-19-related changes in total and anti-S IgG_1_ glycosylation profiles are not correlated with changes in CRP levels. Contrarily, changes in anti-S IgG_1_ galactosylation and sialylation were both found to significantly correlate with changes in plasma anti-S IgG concentration recorded between the last and the first time-point of patients’ hospital stay (Table 2).

**TABLE 2:** Correlation between COVID-19-related differences in total and anti-S IgG_1_-specific glycosylation traits and the difference in CRP and anti-S IgG plasma concentration in COVID-19 patients.

		Total IgG_1_				Anti-S IgG_1_			
		ΔFuc	ΔGal	ΔSial	ΔBisec	ΔFuc	ΔGal	ΔSial	ΔBisec
ΔCRP	*r*	−0.217	0.246	0.032	−0.351	0.166	0.311	0.218	−0.176
	*p*	1	1	1	0.8	1	1	1	1
Δanti-S IgG	*r*	−0.123	−0.194	−0.033	−0.332	−0.175	−0.717	−0.617	−0.075
	*p*	1	1	1	0.784	1	0.0016	0.0064	1

*The difference between the last and the first time-point of hospitalization (Δ) was calculated for each parameter or glycosylation trait according to the formula: ΔX = X last − X first. For all glycosylation traits, descriptive statistics are shown in terms of r (Pearson’s correlation coefficient) and p (p-values). The presented p-values are Bonferroni-adjusted, statistical significance was reached when p < 0.05. Representations of glycosylation traits are given in terms of Fuc (fucosylation), Gal (galactosylation), Sial (sialylation), and Bisec (bisecting GlcNAc).*

## Discussion

An increasing number of evidences suggest that altered IgG glycosylation might be a factor contributing to disease severity in COVID-19. To further deepen the understanding of molecular signatures underlying the SARS-CoV-2 infection, in this work, we performed a longitudinal analysis of total and anti-S IgG Fc-glycosylation in a cohort of 35 hospitalized COVID-19 patients and 35 HC by means of MALDI-TOF-MS. To assure site- and subclass-specificity of determined glycosylation profiles, in this study, all analyses were performed at the level of tryptic glycopeptides.

Upon SARS-CoV-2 infection, virus-specific IgG antibodies are typically detected in blood within 7 days from symptom onset (Long et al., 2020; Dan et al., 2021; Pang et al., 2021; Semmler et al., 2021; Sterlin et al., 2021). In our study, the first detection of SARS-CoV-2-specific anti-S glycopeptides in plasma of affected patients occurred between the hospitalization day 0 and day 6. While this broad range might partly result from the differences in patients’ hospital admission time, it might as well reflect the inter-individual variability of the humoral immune response to SARS-CoV-2 infection.

In our study, COVID-19 was found to be associated with significant changes in total and antigen-specific IgG glycosylation. In particular, anti-S IgG_1_ produced in the early stage of the disease was found to be marked by strongly decreased core-fucosylation, which is in line with previously reported data (Hoepel et al., 2021; Larsen et al., 2021). Of note, this particular feature of SARS-CoV-2-specific antibodies has important functional consequences. Precisely, although afucosylation of the IgG Fc portion does not influence the binding toward viral particles, it enhances by several folds the binding affinity toward FcγRIIIa receptors on the surface of innate immune cells (Wang et al., 2017; Chakraborty et al., 2021). These Fc-FcγR interactions are crucial for Fc-mediated effector functions such as antibody-dependent cellular cytotoxity and phagocytosis, which next to viral neutralization are the primarily mechanisms contributing to anti-viral host protection (Taylor et al., 2021). Data reported by Bye et al. (2021) indicate, however, that low fucosylation of anti-SARS-CoV-2 Spike IgG might be a double-edged sword; while it facilitates the recovery process by potentiating anti-viral immune responses, it might contribute to COVID-19 patients’ death by enhancing pathogenic platelet activation and thrombosis (Bye et al., 2021). In line with this data, immune complexes engaging afucosylated IgG molecules were shown to stimulate the expression of pro-inflammatory cytokines (e.g., IL-6, TNF, and IL-1β) in macrophages and natural killer cells, generating a prothrombotic environment (Chakraborty et al., 2021; Larsen et al., 2021). In addition, high titers and low fucosylation of anti-S IgG_1_ were recently shown to promote inflammation by alveolar macrophages (Hoepel et al., 2021).

Consistent with data of Larsen et al. (2021), in this work, the initially low fucosylation of antigen-specific IgG_1_ was found to increase continuously over time, eventually approaching the levels observed in total IgG_1_. It seems plausible that this timely restricted production of highly potent afucosylated anti-viral antibodies helps preventing excessive and potentially harmful immune activation. Contrary to the above described findings, in the study of Chakraborty et al. (2021), afucosylation levels were shown to be stable over time. This discrepancy might be caused by the fact that, in the latter study, the investigated samples were not collected at the onset of anti-S IgG_1_ expression.

Interestingly, some reports indicate that low fucosylation of antigen-specific IgG antibodies might as well play a role in other acute viral infections. For instance, a longitudinal study of dengue-infected patients showed that, at the early stage of the disease, IgG glycosylation profile is marked by high afucosylation, which decreases in the course of the disease the way we have measured here for anti-S IgG (Wang et al., 2017). Contrarily, antibodies against internal viral proteins such as nucleocapsid in COVID-19 or the parvovirus B19 were not afucosylated but rather highly fucosylated (Larsen et al., 2021). These seemingly contradictory data suggest that the biological function of IgG fucosylation might differ depending on the nature of the infectious agent, but this has not been investigated in details so far.

Besides profiling the COVID-19-related IgG glycosylation changes, the aim of our study was to determine whether observed alterations are associated with patients age and whether they correlate with changes in CRP and anti-SARS-CoV-2 IgG plasma concentrations. Notably, for the vast majority of conducted analyses, statistically significant trends were observed exclusively in antigen-specific anti-S IgG_1_, which further confirms that the glycosylation of bulk and anti-viral IgG in COVID-19 might be distinctly regulated. In this work, anti-S IgG_1_ fucosylation was the only glycosylation trait whose change was correlated with patients’ age during hospitalization. Precisely, in the course of the disease, anti-S IgG_1_ antibodies of younger COVID-19 patients displayed a more prominent alteration in the fucosylation level as compared to older patients. In the light of what has been written above, it seems plausible that this dynamic timely restricted transition from highly pro-inflammatory afucosylated phenotype at seroconversion to less pro-inflammatory fucosylated anti-S IgG_1_ phenotype observed later in the disease course comprises an innate regulatory mechanism that allows younger individuals to mount a more potent virus-specific immune response that is limited to the early phase of the disease. Following this understanding, a weaker change in anti-S IgG_1_ fucosylation observed in older COVID-19 patients could contribute to compromised or less balanced anti-viral response. In line with this rationale, the quality of the humoral response was shown to decline with age, which was linked to diminished potential of aged B-cells to undergo somatic hypermutations (Frasca et al., 2017). Correspondingly, the quantity and glycosylation profile of anti-SARS-CoV-2 antibodies elicited in response to COVID-19 mRNA vaccination were shown to differ in younger and older individuals (Farkash et al., 2021). In line with our data, anti-SARS-CoV-2 antibodies produced after the first and the second vaccination showed higher variation with respect to the fucosylation level in younger as opposed to older individuals (Farkash et al., 2021). Interestingly, in our study, no correlation was observed between the change in anti-S IgG_1_ fucosylation and the change in anti-SARS-CoV-2 antibody titer in the course of hospitalization, implying that the above-described age-dependent transformation of IgG fucosylation in COVID-19 patients is independent of anti-S IgG abundancy in blood. Nevertheless, considering that the vast majority of the investigated cohort was represented by severe COVID-19 patients, who presented overall high anti-Spike IgG titers, the latter observation necessitates validation in a larger and more diversified cohort.

In line with the results of Hoepel et al. (2021) and Larsen et al. (2021), anti-S IgG_1_ antibodies investigated in the present study were marked by high galactosylation and sialylation as compared to their corresponding levels in total IgG_1_. Particularly, this glycosylation profile of anti-SARS-CoV-2 antibodies was specific to the early stage of the disease, as the disparity between total and antigen-specific IgG galactosylation/sialylation diminished continuously toward the end of hospitalization. Notably, this profile was shown to be associated with previous natural infection and recent immunization (Wang et al., 2015; Sonneveld et al., 2017; Hoepel et al., 2021). Interestingly, contrary to the trends observed for fucosylation, COVID-19-related decrease in anti-S IgG_1_ galactosylation and sialylation showed no correlation with patients’ age; instead, they were found to negatively correlate with changes in anti-S IgG plasma concentration. Considering that, along with previous reports (Hoepel et al., 2021; Larsen et al., 2021), anti-S IgG_1_ concentration and anti-S IgG_1_ galactosylation/sialylation levels were, respectively, observed to increase and decrease in the course of the disease, the above findings imply that a strong increase in anti-S IgG concentration, reported to occur in severe COVID-19 patients (Larsen et al., 2021; Madariaga et al., 2021), might be accompanied with less prominent decrease in anti-S IgG_1_ galactosylation and sialylation. Notably, the persistence of highly galactosylated/sialylated anti-S IgG_1_ antibodies that are otherwise limited to acute, early stage of the disease, could potentially contribute to the severity of the disease. This observation is advocated by the fact that platelet-mediated thrombosis that contributes to increased mortality in critically ill COVID-19 patients was shown to require both low levels of fucosylation and high levels of galactosylation in the anti-S IgG Fc domain (Bye et al., 2021).

Expectedly, inflammation accompanying SARS-CoV-2 infection was reflected by strong CRP elevation in COVID-19 patients at the beginning of hospitalization. Afterward, in the COVID-19 patients who survived the disease, CRP levels were found to gradually decrease in the disease course, with the biggest change being observed during the first half of hospitalization. In our study, the difference in CRP level showed no correlation with changes observed in IgG glycosylation traits.

It should be noted that the present study suffers from some limitations. First and foremost, the analyses were conducted on a relatively small number of samples, with COVID-19 cohort consisting predominantly of patients having severe disease symptoms. Therefore, it would be meaningful to validate these findings in a larger and more diversified cohort. Additionally, anti-S IgG fractions investigated in our study contained exclusively IgG_1_ glycopeptides, as antibodies of this subclass dominate the immune response directed toward S1 subunits of the viral Spike protein (Yates et al., 2021). It would be of interest to determine whether reported trends are as well observed in IgG_3_, whose titers were recently reported to increase upon SARS-CoV-2 infection, particularly in response to SARS-CoV-2 Spike S2 subunit (Luo et al., 2021; Yates et al., 2021).

In conclusion, the results presented in this study confirm previous findings showing that anti-S IgG_1_ antibodies produced in COVID-19 patients are marked by differential glycosylation profiles, which normalize gradually in the course of the disease. Using a German cohort, we were able to show that COVID-19-related glycosylation alterations that occur in antigen-specific anti-S antibodies are to some extend dependent on patient’s age and anti-S IgG quantity. Further studies are needed to determine whether these observed trends are specific to anti-Spike S1 subunit IgG_1_ antibodies and whether they are likewise detected in COVID-19 patients suffering from less severe disease symptoms.

## Data Availability Statement

The raw data supporting the conclusions of this article will be made available by the authors, without undue reservation.

## Ethics Statement

The studies involving human participants were reviewed and approved by the Charité – Universitätsmedizin Berlin, Campus Virchow-Klinikum, Germany (no. EA2/095/20) and by the Ärztekammer Berlin, Germany (Eth-23/20). All experiments for EA2/095/20 were performed in accordance with relevant guidelines and regulations. Additional written informed consent was taken for Eth-23/20.

## Author Contributions

VB contributed to the conception and design of the study. KK, BH, JR, and GH coordinated the collection of samples and database. CS and MG performed the experiments and data analysis. VB, MG, and CS wrote the manuscript. All authors contributed to manuscript revision, read, and approved the submitted version.

## Conflict of Interest

KK is partly contractually provided to Labor Berlin – Charité Vivantes GmbH. The remaining authors declare that the research was conducted in the absence of any commercial or financial relationships that could be construed as a potential conflict of interest.

## Publisher’s Note

All claims expressed in this article are solely those of the authors and do not necessarily represent those of their affiliated organizations, or those of the publisher, the editors and the reviewers. Any product that may be evaluated in this article, or claim that may be made by its manufacturer, is not guaranteed or endorsed by the publisher.

## Supplementary Material

Supplementary Figure 1 Inter-day repeatability testing of the analytical workflow used in this study. Average is shown as mean ± SD.

Supplementary Figure 2 Coronavirus disease 2019-related glycosylation alterations in patients with shorter (<20 days) and longer (≥20 days) hospitalization. **(A)** Total IgG_1_, **(B)** anti-S IgG_1_, and **(C)** total IgG_2_. The calculation of the glycosylation traits Gal, Bisec, Sial, and Fuc is given in section “MALDI-TOF Measurements and Data Analysis.” **p* < 0.05, ***p* < 0.01.

Supplementary Figure 3 Longitudinal changes of (left) anti-S IgG_1_ and (right) total IgG_1_ Fc glycosylation in COVID-19 patients; **(A,B)** fucosylation, **(C,D)** galactosylation, **(E,F)** sialylation, and **(G,H)** bisecting GlcNAc. Data points corresponding to the two deceased COVID-19 patients are indicated in red (patient 1) and blue (patient 2).

Supplementary Figure 4 The age-related IgG glycosylation changes in both healthy controls (HC) and COVID-19 patients represented with the two major IgG_1_ *N*-glycopeptide structures, namely **(A)** agalactosylated G0F and **(B)** digalactosylated G2F.

Supplementary Figure 5 Plasma CRP and anti-S IgG concentration in COVID-19 patients. **(A,D)** CRP and anti-S IgG levels at the beginning, in the middle, and at the end of patients’ hospital stay. **(B,E)** Longitudinal changes in CRP and anti-S IgG_1_ levels in the course of the disease. **(C,F)** Correlation of CRP and anti-S IgG levels with patients’ age; the *Y* axes represent the difference (Δ) in the respective parameter recorded between the last and first hospitalization time-point. Data points corresponding to the two deceased COVID-19 patients are indicated in red (patient 1) and blue (patient 2). For patient 1, only one measurement of CRP was performed. ***p* < 0.01, ****p* < 0.001.
