# Supplementary material for: Coronavirus Disease 2019-Related Alterations of Total and Anti-Spike IgG Glycosylation in Relation to Age and Anti-Spike IgG Titer
Source: Front Microbiol. 2022 Apr 15;13:775186. doi: 10.3389/fmicb.2022.775186 (PMC9051488; doi:10.3389/fmicb.2022.775186)
Supplement: Supplementary file 1 [file Table_1.DOCX]

|  | | *m/z* [M-H]^-^ of detected glycoforms (Da) | |
| --- | --- | --- | --- |
| Structure composition | | IgG_1_  (EEQYNSTYR) | IgG_2_  (EEQFNSTFR) |
| G0F |  | 2632.04 | 2600.04 |
| G1F |  | 2794.09 | 2762.09 |
| G2F |  | 2956.14 | 2924.15 |
| G0FN |  | 2835.12 | 2803.12 |
| G1FN |  | 2997.17 | 2965.17 |
| G2FN |  | 3159.22 | 3127.23 |
| G1FS1 |  | 3085.18 | 3053.19 |
| G2FS1 |  | 3247.24 | 3215.24 |
| Mono G0F |  | 2428.96 | - |
| Mono G1F |  | 2591.01 | - |
| G0 |  | 2485.98 | 2453.98 |
| G1 |  | 2648.03 | -* |
| G2 |  | 2810.08 | -* |
| G0N |  | 2689.06 | 2657.06 |
| G1N |  | 2851.11 | -* |
| G2N |  | 3013.16 | -* |
| G1S1 |  | - | 2907.13 |
| G2S1 |  | 3101.18 | -* |

**Table S1.** Tryptic glycopeptides of human IgG1 and IgG2 measured by MALDI-TOF-MS in this study. Schematic representations are given in terms of pink diamond (sialic acid), yellow circle (galactose), blue square (N-acetylglucosamine), green circle (mannose), red triangle (fucose), pep (peptide moiety). Structural composition is given in terms of G (galactose), F (fucose), N (bisecting GlcNAc), S (sialic acid), Mono (monoantennary). * isomeric glycopeptide species of IgG2 and IgG4.
