# Supplementary material for: Coronavirus Disease 2019-Related Alterations of Total and Anti-Spike IgG Glycosylation in Relation to Age and Anti-Spike IgG Titer
Source: Front Microbiol. 2022 Apr 15;13:775186. doi: 10.3389/fmicb.2022.775186 (PMC9051488; doi:10.3389/fmicb.2022.775186)
Supplement: Supplementary file 2 [file Table_2.DOCX]

| IgG  structure | IgG subclass | Healthy  [Mean ± SD] | COVID-19 (first)  [Mean ± SD] | COVID-19 (middle)  [Mean ± SD] | COVID-19 (last)  [Mean ± SD] |
| --- | --- | --- | --- | --- | --- |
| G0F | Total IgG_1_ | 27.8 ± 9.0 | 34.1 ± 10.2 | 38.9 ± 11.8 | 41.1 ± 13.8 |
|  | anti-S IgG_1_ | - | 14.8 ± 7.0 | 24.2 ± 11.4 | 28.7 ± 16.2 |
|  | Total IgG_2_ | 43.2 ± 10.7 | 52.2 ± 12.1 | 55.3 ± 12.1 | 57.0 ± 13.5 |
| G1F | Total IgG_1_ | 38.8 ± 6.4 | 42.0 ± 6.5 | 40.4 ± 7.6 | 38.9 ± 8.4 |
|  | anti-S IgG_1_ | - | 39.8 ± 9.9 | 44.8 ± 6.7 | 42.1 ± 9.3 |
|  | Total IgG_2_ | 34.8 ± 7.1 | 33.5 ± 8.7 | 31.8 ± 8.7 | 30.2 ± 9.4 |
| G2F | Total IgG_1_ | 12.3 ± 5.4 | 9.9 ± 4.6 | 8.9 ± 4.4 | 8.5 ± 5.0 |
|  | anti-S IgG_1_ | - | 17.4 ± 7.4 | 12.9 ± 5.2 | 12.9 ± 6.1 |
|  | Total IgG_2_ | 7.3 ± 4.4 | 4.9 ± 3.1 | 4.3 ± 2.7 | 4.1 ± 3.0 |
| G0FN | Total IgG_1_ | 4.6 ± 2.3 | 3.3 ± 2.1 | 2.9 ± 2.0 | 3.1 ± 2.3 |
|  | anti-S IgG_1_ | - | 0.1 ± 0.2 | 0.7 ± 0.9 | 0.9 ± 1.4 |
|  | Total IgG_2_ | 5.9 ± 2.4 | 4.7 ± 2.1 | 4.0 ± 1.8 | 4.1 ± 2.0 |
| G1FN | Total IgG_1_ | 6.7 ± 2.6 | 3.8 ± 1.7 | 3.0 ± 2.0 | 3.0 ± 1.6 |
|  | anti-S IgG_1_ | - | 1.0 ± 1.2 | 1.3 ± 1.2 | 1.1 ± 1.0 |
|  | Total IgG_2_ | 3.0 ± 1.4 | 1.6 ± 0.8 | 1.4 ± 0.7 | 1.4 ± 0.8 |
| G2FN | Total IgG_1_ | 0.7 ± 0.3 | 0.2 ± 0.1 | 0.2 ± 0.1 | 0.2 ± 0.1 |
|  | anti-S IgG_1_ | - | - | - | 0.1 ± 0.1 |
|  | Total IgG_2_ | 0.4 ± 0.3 | 0.1 ± 0.2 | 0.1 ± 0.1 | 0.1 ± 0.1 |
| G1FS1 | Total IgG_1_ | 0.5 ± 0.2 | 0.4 ± 0.1 | 0.3 ± 0.1 | 0.3 ± 0.2 |
|  | anti-S IgG_1_ | - | 0.5 ± 0.7 | 0.9 ± 0.8 | 0.8 ± 0.8 |
|  | Total IgG_2_ | 2.0 ± 0.7 | 1.4 ± 0.6 | 1.4 ± 0.6 | 1.4 ± 0.8 |
| G2FS1 | Total IgG_1_ | 2.0 ± 1.0 | 1.8 ± 1.1 | 1.6 ± 1.2 | 1.7 ± 1.4 |
|  | anti-S IgG_1_ | - | 9.7 ± 6.4 | 7.4 ± 5.0 | 6.9 ± 4.6 |
|  | Total IgG_2_ | 1.9 ± 1.3 | 1.3 ± 0.9 | 1.2 ± 0.9 | 1.2 ± 1.0 |
| Mono G0F | Total IgG_1_ | 0.1 ± 0.1 | 0.1 ± 0.1 | 0.1 ± 0.1 | 0.1 ± 0.1 |
|  | anti-S IgG_1_ | - | 0.1 ± 0.2 | 0.1 ± 0.3 | 0.1 ± 0.2 |
|  | Total IgG_2_ | - | - | - | - |
| Mono G1F | Total IgG_1_ | 0.1 ± 0.2 | 0.1 ± 0.2 | 0.1 ± 0.2 | 0.1 ± 0.2 |
|  | anti-S IgG_1_ | - | 0.1 ± 0.1 | 0.1 ± 0.1 | 0.1 ± 0.2 |
|  | Total IgG_2_ | - | - | - | - |
| G0 | Total IgG_1_ | 0.7 ± 0.7 | 0.9 ± 1.0 | 0.9 ± 1.0 | 0.8 ± 0.9 |
|  | anti-S IgG_1_ | - | 3.5 ± 2.8 | 2.1 ± 2.1 | 1.7 ± 1.9 |
|  | Total IgG_2_ | 0.2 ± 0.2 | 0.1 ± 0.1 | 0.1 ± 0.1 | 0.1 ± 0.1 |
| G1 | Total IgG_1_ | 2.2 ± 1.5 | 1.8 ± 1.5 | 1.6 ± 1.1 | 1.3 ± 1.0 |
|  | anti-S IgG_1_ | - | 8.8 ± 7.3 | 3.8 ± 4.3 | 3.2 ± 5.7 |
|  | Total IgG_2_ | - | - | - | - |
| G2 | Total IgG_1_ | 1.1 ± 0.7 | 0.7 ± 0.6 | 0.6 ± 0.4 | 0.5 ± 0.4 |
|  | anti-S IgG_1_ | - | 3.6 ± 4.0 | 1.4 ± 2.6 | 1.0 ± 2.5 |
|  | Total IgG_2_ | - | - | - | - |
| G0N | Total IgG_1_ | 0.6 ± 0.4 | 0.2 ± 0.1 | 0.2 ± 0.1 | 0.2 ± 0.1 |
|  | anti-S IgG_1_ | - | 0.1 ± 0.3 | 0.1 ± 0.2 | 0.2 ± 0.3 |
|  | Total IgG_2_ | 1.3 ± 1.1 | 0.3 ± 0.3 | 0.5 ± 0.6 | 0.5 ± 0.4 |
| G1N | Total IgG_1_ | 1.1 ± 0.6 | 0.3 ± 0.2 | 0.3 ± 0.1 | 0.3 ± 0.1 |
|  | anti-S IgG_1_ | - | 0.1 ± 0.3 | 0.3 ± 0.5 | 0.2 ± 0.3 |
|  | Total IgG_2_ | - | - | - | - |
| G2N | Total IgG_1_ | 0.5 ± 0.3 | 0.1 ± 0.1 | 0.1 ± 0.1 | 0.1 ± 0.1 |
|  | anti-S IgG_1_ | - | 0.1 ± 0.1 | 0.1 ± 0.1 | 0.1 ± 0.1 |
|  | Total IgG_2_ | - | - | - | - |
| G1S1 | Total IgG_1_ | - | - | - | - |
|  | anti-S IgG_1_ | - | - | - | - |
|  | Total IgG_2_ | 0.1 ± 0.2 | - | - | - |
| G2S1 | Total IgG_1_ | 0.2 ± 0.2 | 0.1 ± 0.1 | 0.1 ± 0.1 | 0.1 ± 0.1 |
|  | anti-S IgG_1_ | - | 0.7 ± 1.3 | 0.1 ± 0.3 | 0.1 ± 0.4 |
|  | Total IgG_2_ | - | - | - | - |
| Gal | Total IgG_1_ | 41.3 ± 8.3 | 37.0 ± 8.6 | 34.2 ± 9.2 | 32.9 ± 10.7 |
|  | anti-S IgG_1_ | - | 56.4 ± 10.0 | 47.3 ± 10.8 | 44.6 ± 13.5 |
|  | Total IgG_2_ | 39.7 ± 11.5 | 24.6 ± 8.3 | 22.8 ± 7.8 | 21.8 ± 8.8 |
| Sial | Total IgG_1_ | 14.2 ± 4.8 | 2.3 ± 1.2 | 2.0 ± 1.4 | 2.1 ± 1.6 |
|  | anti-S IgG_1_ | - | 10.9 ± 6.2 | 8.4 ± 5.2 | 7.8 ± 4.9 |
|  | Total IgG_2_ | 4.0 ± 1.8 | 2.7 ± 1.4 | 2.6 ± 1.5 | 2.6 ± 1.8 |
| Bisec | Total IgG_1_ | 2.7 ± 1.2 | 7.9 ± 3.5 | 6.5 ± 3.0 | 6.7 ± 3.3 |
|  | anti-S IgG_1_ | - | 1.3 ± 1.6 | 2.3 ± 2.0 | 2.5 ± 2.2 |
|  | Total IgG_2_ | 10.6 ± 4.1 | 6.8 ± 2.6 | 5.9 ± 2.1 | 6.1 ± 2.4 |
| Fuc | Total IgG_1_ | 93.7 ± 3.6 | 95.9 ± 3.1 | 96.4 ± 2.5 | 96.8 ± 2.3 |
|  | anti-S IgG_1_ | - | 83.3 ± 14.2 | 92.3 ± 7.9 | 93.5 ± 9.5 |
|  | Total IgG_2_ | - | - | - | - |

**Table S2.** Mean relative abundancies and SD values of IgG glycosylation peaks in healthy controls and COVID-19 patients. Structural composition is given in terms of G (galactose), F (fucose), N (bisecting GlcNAc), S (sialic acid), Mono (monoantennary). Representations of glycosylation traits are given in terms of Gal (galactosylation), Sial (sialylation), Bisec (bisecting GlcNAc), Fuc (core-fucosylation) as defined in section 2.6.
