# Supplementary figures and images for: Coronavirus Disease 2019-Related Alterations of Total and Anti-Spike IgG Glycosylation in Relation to Age and Anti-Spike IgG Titer
Source: Front Microbiol. 2022 Apr 15;13:775186. doi: 10.3389/fmicb.2022.775186 (PMC9051488; doi:10.3389/fmicb.2022.775186)

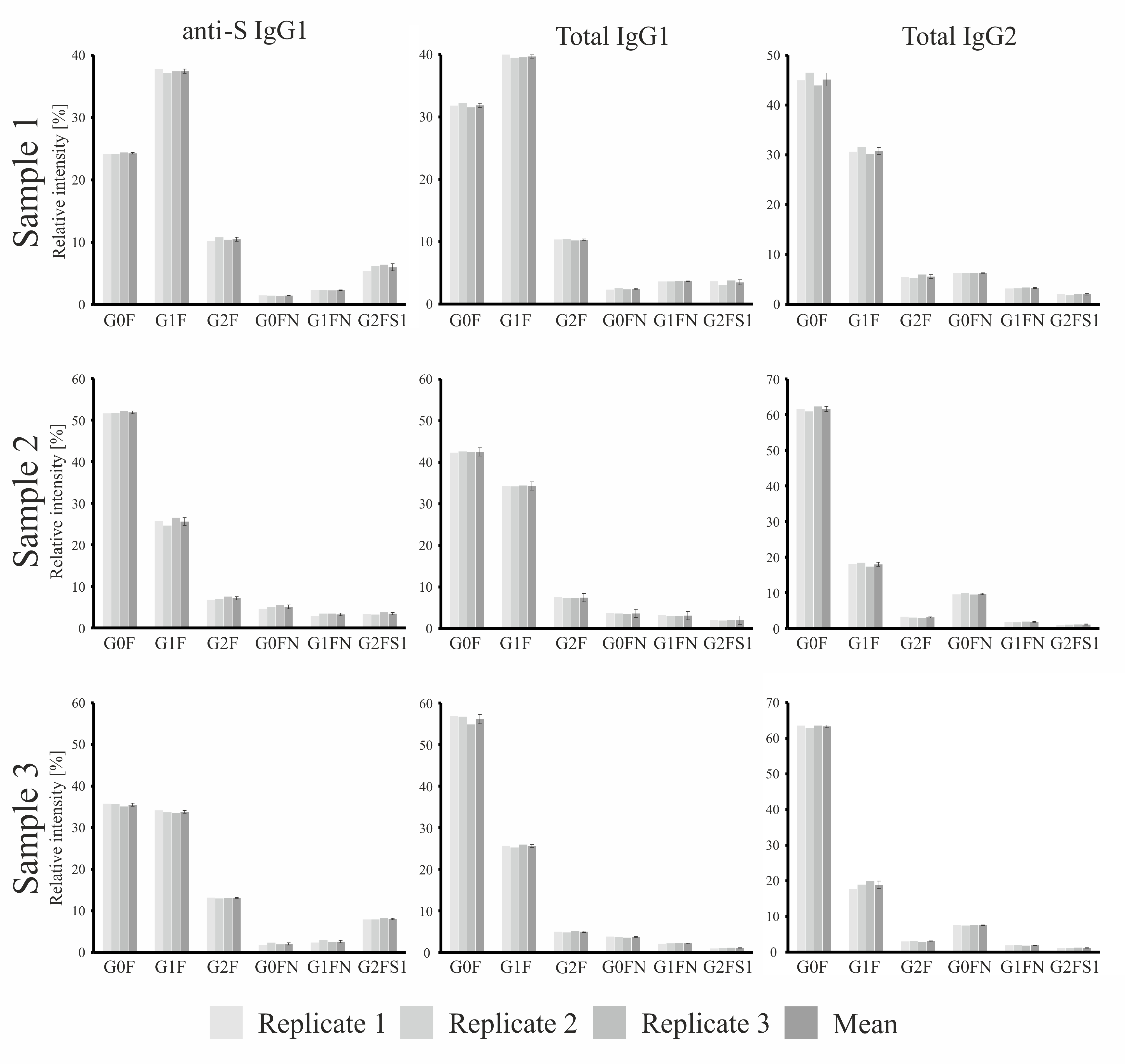

Supplement: Supplementary Figure 1 — Inter-day repeatability testing of the analytical workflow used in this study. Average is shown as mean ± SD. [file Image_1.JPEG]

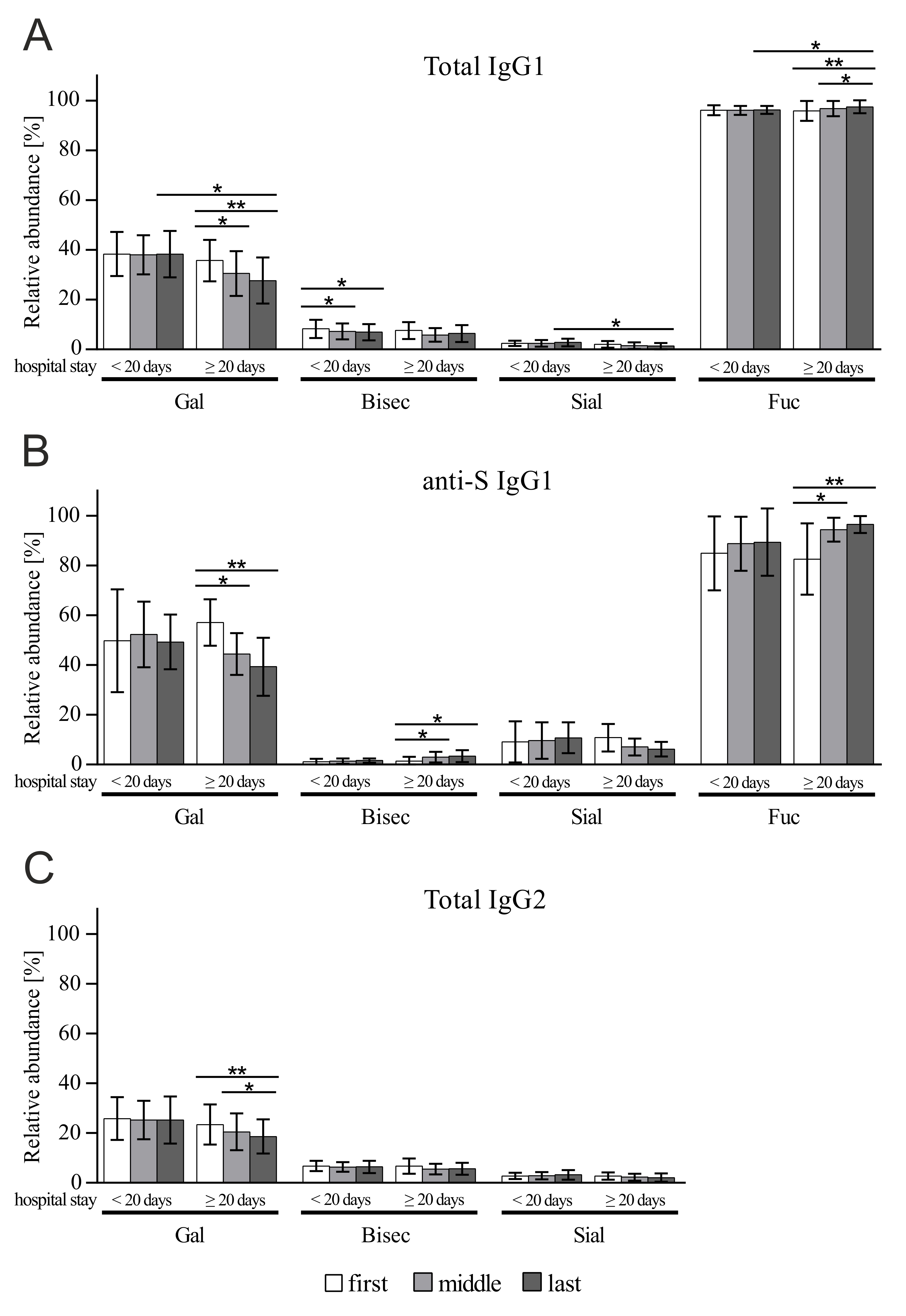

Supplement: Supplementary Figure 2 — Coronavirus disease 2019-related glycosylation alterations in patients with shorter (<20 days) and longer (≥20 days) hospitalization. (A) Total IgG1, (B) anti-S IgG1, and (C) total IgG2. The calculation of the glycosylation traits Gal, Bisec, Sial, and Fuc is given in section “MALDI-TOF Measurements and Data Analysis.” *p < 0.05, **p < 0.01. [file Image_2.JPEG]

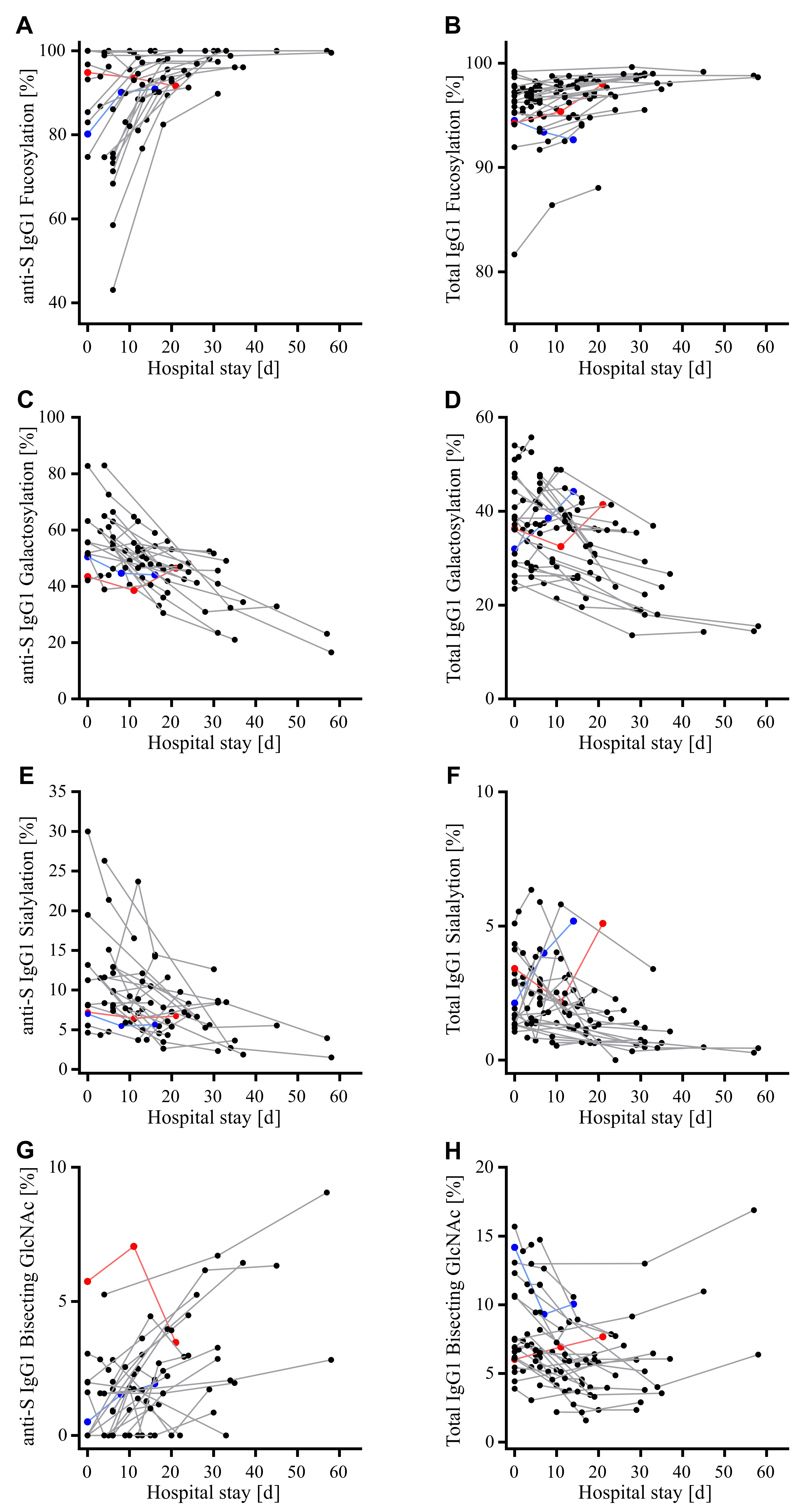

Supplement: Supplementary Figure 3 — Longitudinal changes of (left) anti-S IgG1 and (right) total IgG1 Fc glycosylation in COVID-19 patients; (A,B) fucosylation, (C,D) galactosylation, (E,F) sialylation, and (G,H) bisecting GlcNAc. Data points corresponding to the two deceased COVID-19 patients are indicated in red (patient 1) and blue (patient 2). [file Image_3.JPEG]

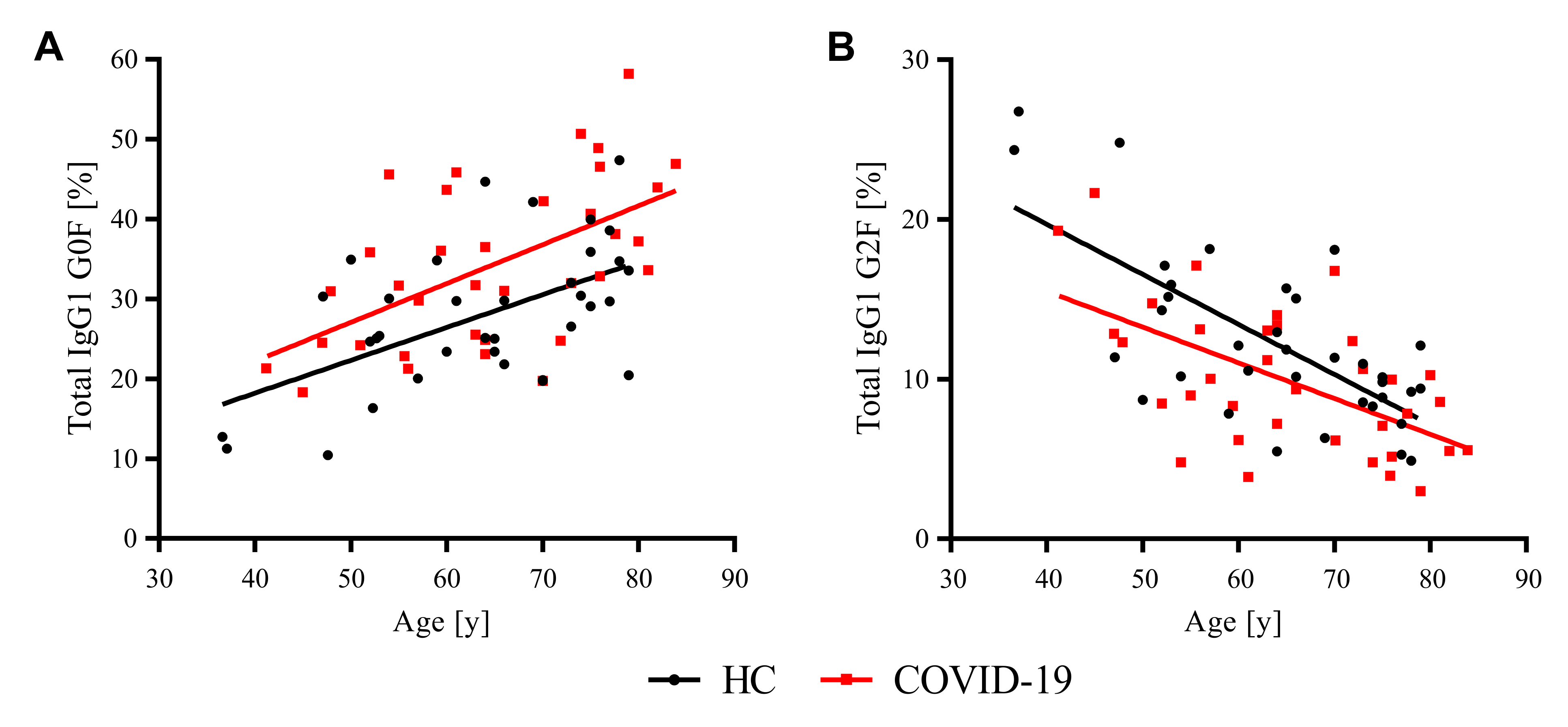

Supplement: Supplementary Figure 4 — The age-related IgG glycosylation changes in both healthy controls (HC) and COVID-19 patients represented with the two major IgG1 N-glycopeptide structures, namely (A) agalactosylated G0F and (B) digalactosylated G2F. [file Image_4.JPEG]

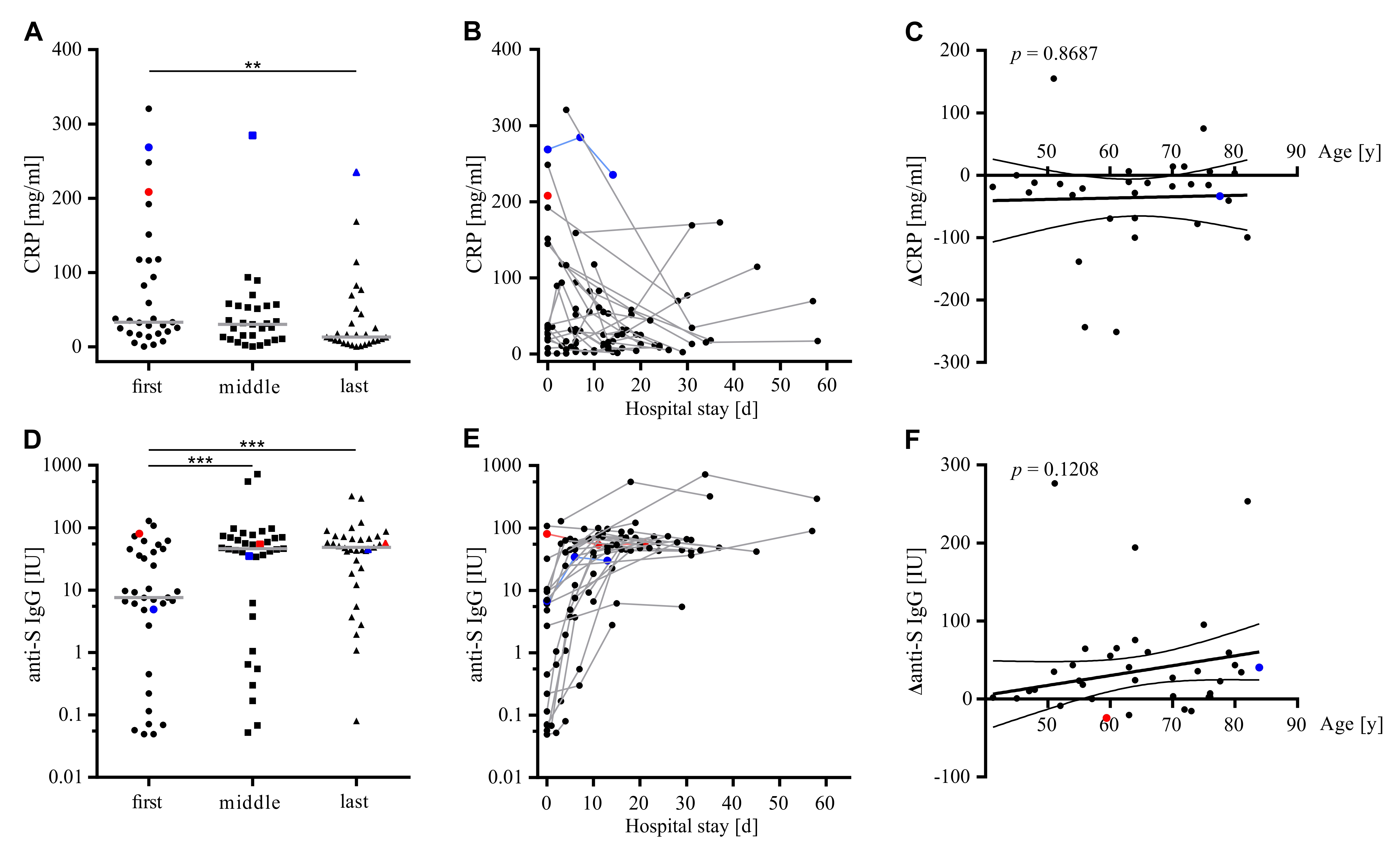

Supplement: Supplementary Figure 5 — Plasma CRP and anti-S IgG concentration in COVID-19 patients. (A,D) CRP and anti-S IgG levels at the beginning, in the middle, and at the end of patients’ hospital stay. (B,E) Longitudinal changes in CRP and anti-S IgG1 levels in the course of the disease. (C,F) Correlation of CRP and anti-S IgG levels with patients’ age; the Y axes represent the difference (Δ) in the respective parameter recorded between the last and first hospitalization time-point. Data points corresponding to the two deceased COVID-19 patients are indicated in red (patient 1) and blue (patient 2). For patient 1, only one measurement of CRP was performed. **p < 0.01, ***p < 0.001. [file Image_5.JPEG]
